# Luminal B breast cancer subtype displays a dicotomic epigenetic pattern

**DOI:** 10.1186/s40064-016-2235-0

**Published:** 2016-05-14

**Authors:** Naiara G. Bediaga, Elena Beristain, Borja Calvo, María A. Viguri, Borja Gutierrez-Corres, Ricardo Rezola, Irune Ruiz-Diaz, Isabel Guerra, Marian M. de Pancorbo

**Affiliations:** BIOMICs Research Group, Lascaray Research Center, University of the Basque Country (UPV/EHU), Avda. Miguel de Unamuno, 3, 01006 Vitoria-Gasteiz, Spain; Molecular (Epi)Genetics Laboratory, BioAraba National Health Institute, OSI Araba University Hospital, 01009 Vitoria-Gasteiz, Spain; Intelligent Systems Group, University of the Basque Country (UPV/EHU), Donostia-San Sebastián, Basque Country Spain; Service of Anatomic Pathology, OSI Araba University Hospital, 01009 Vitoria-Gasteiz, Spain; Service of Anatomic Pathology, Onkologikoa, Paseo Dr. Begiristain 121, 20014 Donostia-San Sebastián, Gipuzkoa Spain; Service of Anatomic Pathology, Hospital Universitario Donostia, Paseo Dr. Begiristain 107-115, 20014 Donostia-San Sebastián, Gipuzkoa Spain

**Keywords:** Epigenetic, Methylation, Breast cancer, Luminal subtypes

## Abstract

Luminal B breast tumors have aggressive clinical and biological features, and constitute the most heterogeneous molecular subtype, both clinically and molecularly. Unfortunately, the immunohistochemistry correlate of the luminal B subtype remains still imprecise, and it has now become of paramount importance to define a classification scheme capable of segregating luminal tumors into clinically meaningful subgroups that may be used clinically to guide patient management. With the aim of unraveling the DNA methylation profiles of the luminal subtypes currently being most used in the clinical setting, we have quantified the DNA methylation level of 27,578 CpG sites in 17 luminal B (ER+, Ki67 ≥ 20 % or PgR < 20 % and HER2−), 8 luminal A (ER+ and Ki67 > 20 %) and 4 luminal B-HER2+ (ER+ and HER2+) breast cancer samples by using the Illumina Infinium methylation microarray approach. Unsupervised hierarchical clustering revealed that DNA methylation stratifies luminal B samples in two categories with differing epigenetic and clinical features. One subgroup of luminal B samples showed a methylator phenotype and clustered with the lumB-HER tumors, while the other showed less methylated events, clustered with the luminal A. A 3 CpG marker panel capable of discriminating methylator versus non-methylator luminal B samples was identified and further validated in an independent cohort of patients. Our results provide evidence that DNA methylation and, more specifically, a panel of 3 CpG markers, enables the stratification of luminal B samples in two categories with differing epigenetic and clinical features and support the utilization of this panel for therapeutic stratification of patients with luminal breast cancer.

**Electronic supplementary material:**

The online version of this article (doi:10.1186/s40064-016-2235-0) contains supplementary material, which is available to authorized users.

## Background

Breast cancer (BC) is a complex and heterogeneous disease which includes several subtypes with different molecular and clinical characteristics. A major milestone on the classification of the breast carcinomas is the so called “intrinsic classification”, which divides breast tumors into at least five clinically and biologically relevant intrinsic molecular subtypes based on genome-wide expression analyses: luminal A, luminal B, HER2-enriched, basal-like, and normal breast-like. In this respect, the luminal B subtype constitutes the most heterogeneous group, both clinically and molecularly. In fact, although many of the Luminal B tumors are ER+/HER2−/high Ki-67, expression profiles also classify the ER+/HER2+ tumors as luminal B and these patients receive a different therapy regimen (that incorporates targeted anti-HER2 therapy) compared to other luminal B BC subtypes (Cancer Genome Atlas Network 2012). With respect to the HER2-negative luminal B tumors, they are inherently more aggressive than the luminal A and they require a more aggressive therapy. However, although recent studies have reported that some of the HER2-negative tumors could benefit from anti-HER2 therapy treatment (Pogue-Geile et al. 2013), luminal B tumors are generally treated with a combination of endocrine therapy and chemotherapy, though this approach is not always effective. In view of the clinical/molecular heterogeneity of the luminal B tumors, the 12th St. Gallen International Expert Consensus proposed a new classification system that was further updated in the 13th Consensus by which the luminal BC subtypes were separated into three groups based on the ER, progesterone (PgR), HER2 and Ki-67 status—(1) luminal A-like tumors are ER-positive and HER2-negative with low Ki-67 expression (<20 %) and high PgR levels (≥20 %); (2) luminal B-like (HER2-negative) tumors are ER-positive and HER2-negative with high Ki-67 expression (≥20 %) or with low PgR levels (<20 %); and (3) luminal B-HER2 tumors are ER-positive and HER2-positive regardless PgR or Ki-67 expression. Luminal A disease is stated to require only endocrine therapy, whereas in luminal B disease, chemotherapy should be also considered. Thus, one major challenge in the management of luminal BCs is to discriminate those patients that would benefit from cytotoxic drugs or anti-targeted therapy from those that would not.

The epigenetic transcriptional regulation by DNA methylation and modifications of histones is closely associated with corresponding gene expression in human genome. In addition, DNA methylation profiles have shown to be perturbed in a number of human diseases, including cancer (Esteller 2008; Jones and Takai 2001; Keshet et al. 2006; Suzuki and Bird 2008). Furthermore, many studies suggest that epigenetic changes are involved in the earliest phases of tumorigenesis, contributing to the overexpression of oncogenes and downregulation of tumor suppressor genes. Analogously to transcriptomic profiling, DNA methylation profiling has been used to molecularly classify a number of human malignancies as well as to monitor cancer progression based on tumor-specific methylation signatures (Orlando and Brown 2009).

In BC, specific aberrant methylation patterns have been associated with different BC histologic and molecular subtypes (Dedeurwaerder et al. 2011; Bediaga et al. 2010; Holm et al. 2010; Stefansson et al. 2015; Cornen et al. 2014; Li et al. 2014; Kamalakaran et al. 2011). Particularly, ER−/luminal breast tumors have shown to be characterized by a higher frequency of DNA methylation compared to ER−/basal-like tumors; and within the luminal subgroup, luminal A displayed a substantially lower proportion of DNA methylation marks than the luminal B, although they were quite heterogeneous with regard to the methylation status (Stefansson et al. 2015). Taken all together, these data suggest that DNA methylation profiles may play an important role in the development and progression of distinct breast subtypes. However, most of the epigenome-wide studies published so far are performed on mRNA based subtypes instead of on immunohistochemistry (IHC) based BC subtypes, even if the later are the ones often used to guide the best decision-making in patient treatment as a part of the routine work-up of BC. Therefore, we believe that epigenetically profiling luminal (ER+) tumors that have been stratified with the latest IHC based classification scheme will led to a continuous refinement of the molecular classification of the BC and this could open up potentials for identifying and developing appropriate therapies for these tumor subtypes that sometime defies effective treatment.

## Methods

### Samples

A total of 28 luminal subtype BC samples as well as the adjacent tissue samples of eight were obtained from the Pathology Services of the Hospital Universitario Araba-Txagorritxu (Vitoria-Gasteiz), Oncologikoa (Donostia) and Hospital Universitario Donostia (Donostia), all of them from the Basque Country, in the north of Spain. All breast specimens were reviewed by experienced pathologists. The inclusion criteria were availability of DNA from fresh frozen tumor tissue, tumor size between 2 and 3 cm, histological grade between 2 and 3, and, following the classification proposed in the 13th St Gallen International Breast Cancer Conference in 2013, ER+, any PgR, any Ki-67 and HER2-positive for the luminal B-HER2 subtype; ER+, PgR ≥ 20 %, Ki67 < 20 % and HER2-negative for the Luminal A and ER+, Ki67 ≥ 20 % or PgR < 20 % and HER2-negative for the Luminal B. Eligible patients were postmenopausal women with histologically proven invasive ductal breast carcinoma. Additional data such as nodal involvement were also registered. Ethical approval for the study was obtained from the Txagorritxu Hospital ethic committee (No. 2010-064) and samples were collected according to clinicopathological protocols.

Genomic DNA was extracted using a DNeasy Blood & Tissue Kit (Qiagen, Valencia, USA), and DNA was bisulphite treated using the EZ DNA methylation Kit™ (Zymo Research, USA) following the manufacturer’s protocol.

### DNA methylation profiling

The Infinium HumanMethylation27K (Illumina Inc., CA, USA) was used to assess the methylation profile of 28 luminal subtype BCs, as well as 8 adjacent tissue. The assay interrogates the methylation level of 27,578 CpG sites spanning 14,495 protein coding gene promoters and 110 microRNA gene promoters at single-nucleotide resolution. All data were packaged and deposited in NCBI’s Gene Expression Omnibus (GEO) and are accessible through GEO Series accession number [GEO: GSE73808].

### Computational analysis of the methylation levels

The Illumina Infinium methylation microarray data was processed using the Bioconductor lumi package (Du et al. 2010). First, data were extracted with the Genome Studio™ Methylation Module software. The data went through a quality control (QC) step. The data passing QC step was preprocessed using a color balance adjustment of methylated and unmethylated probe intensities between two color channels using a smooth quantile normalization method. The methylated and unmethylated probe intensities were then background adjusted and normalized using the Quantile method. Differential methylation values were detected by applying the Bayesian moderated *t* test on the normalized matrix of intensity values (M values) (Du et al. 2010; Smyth 2005). In order to recognize CpG sited with both high statistical significance and strong biological effects, we identified differentially methylated CpG-sites based on the following criteria: false-discovery-rate-adjusted (FDR-adjusted) p value <0.05 and an absolute DNA methylation differences >than 0.2. Cluster analysis of the epigenome-wide data was performed using unsupervised hierarchical clustering with the Ward. D2 linkage and Manhattan distance as a measure of similarity as described in Stefansson et al. (Stefansson et al. 2015) (heatmap.2 function implemented in the gplot package for R) on the 1264 CpGs found differentially methylated between breast tumors and normal breast samples (Additional file 1: Table S1). We next used the pvclust package in R to define statistically significant tumor clusters as those showing an AU (approximately unbiased) p value >90 (at least five members). Lists of aberrantly methylated CpGs were further analyzed in order to test for differences in the counting of CpGs by categories reflecting functionally relevant sequences: (1) promoter regions, those located proximal to the transcription start site (TSS) (i.e. CpG’s located within 200 bp of the TSS or within the 5′UTR/1stExon); (2) TSS1500, those located more distantly from the TSS lying within the interval from 200 to 1500 bp upstream of the TSS site; (3) gene body, those found within the gene body (excluding the 1st Exon) and (4) 3′UTR, those located at the 3′UTR. In addition, CpGs were classified as into three different groups, i.e. those found within (1) CpG islands, (2) CpG shores and (3) CpG poor regions. Continuous variables were compared by use of the Mann–Whitney U test and distributions of categorical variables by Chi squared contingency tests. Finally, the predictive power, which measures the ability of a single site to differentiate the samples in each cluster, was evaluated using the area under the ROC curve (AUC) associated to the site, where an AUC of 1 means that the methylation level of that site perfectly separates the samples into the two clusters.

## Results

### Luminal breast cancer subtypes display distinct clinicopathologic characteristics

With the aim of assessing the epigenetic heterogeneity among BC subtypes with similar histologically characteristics, only tumor size between 2 and 3 cm and histological grade between 2 and 3 were included in the current study. Thus, as expected, we did not find any statistical difference in histological grade and tumor size, and neither did we in the nodule involvement. Patient characteristics are presented in Table 1.

**Table 1:** Patient and tumor characteristics

	Subtype							
	Luminal A		Luminal B-Her2		Luminal B		LumB versus LumA	LumB versus LumB-HER2
	Count	Proportion	Count	Proportion	Count	Proportion		
Histological grade								
II	8	1	2	0.5	13	0.76	0.269^a^	0.316^a^
III	0	0	2	0.5	4	0.24		
Tumor size								
<1 cm	3	0.375	1	3	10	0.58	0.659^a^	0.311^a^
≥2 cm	5	0.625	3	8	7	0.42		
Node involvement								
Negative	3	0.42	1	0.25	8	0.47	1^a^	0.603^a^
Positive	4	0.58	3	0.75	9	0.53		
Ki-67								
Mean	10 %		50 %		43 %		0.0001^b^	0.362^b^
SD	4 %		22 %		23 %			
PgR status								
Negative	0	0	1	0.25	10	0.58	0.008^a^	0.311^a^
Positive	8	1	3	0.75	7	0.42		

^a^Chi squared test

^b^Mann–Whitney U test

### Unsupervised clustering of the luminal subtype samples based on DNA methylation profiling

**Fig. 1:**
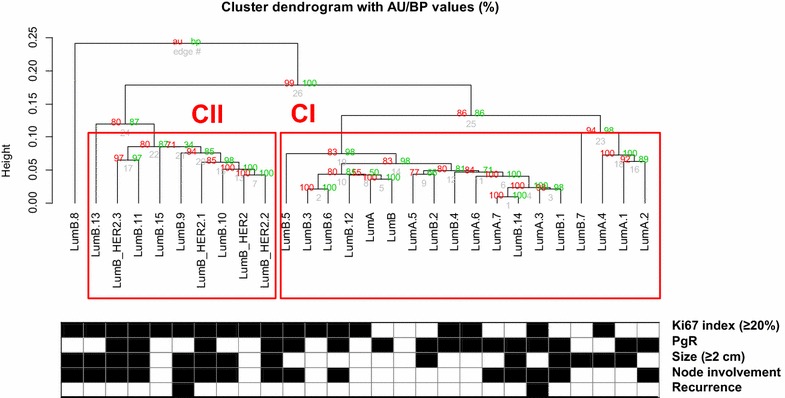
Unsupervised hierarchical clustering of 28 luminal BCs based on 1264 differently methylated CpGs between breast tumors and normal breast. The dendrogram was built with the Manhattan distance as dissimilarity metric and the Ward. 2 linkage method for definition of the structure. Values on the edges of the clustering are p values (%). *Red* values on de egde of the clustering are (Approximately Unbiased) p values. AU values were computed by multiscale bootstrap resampling. R-cran “pvclust” package was used for assessing the robustness of these hierarchical clusters through multiscale bootstrap resampling of the genes

Unsupervised hierarchical clustering based on the list of 1264 differently methylated CpGs between breast tumors and normal breast (Additional file 1: Table S1) identified two significant tumors groups (significant at AU > 95 %; by the pvclust method) (Fig. 1). This classification was compared to the BC luminal subtypes as previously determined by histological profiling. Cluster II (CII) grouped the whole set of luminal B-HER2 and 31.25 % of the luminal B phenotypes. These tumors showed extensive DNA methylation of CpGs implying that they have acquired a methylator phenotype. On the other hand, cluster I (CI) contained the entire set of luminal A phenotypes plus 62.5 % of the luminal B tumors. Overall this cluster showed a lower degree of methylation. When we compared the clinical and histological characteristics between the CI and CII, we found some statistically significant differences. Overall, luminal tumors in CI were smaller (p = 0.01), showed a lower Ki-67 levels (p = 0.01) and probably better outcome (the only two patients with relapsed disease were in CII). Specifically, when only the luminal B samples were selected, we found that luminal B samples fitting in CI were also smaller (p = 0.01) and had a better outcome.

### Identification of cluster specific DNA methylation profiles

In order to identify CpGs with highly significant cluster specific changes, stringent cut-offs (adjusted p value <0.05 and ∆b > 0.20) were set for methylation level changes relative to controls for each cluster. Distinct CpG loci groups containing no known SNPs were identified showing differential methylation profiles in each of the clusters (Additional file 1: Tables S2 and S3). Overall, CII displayed a higher amount of aberrantly methylated CpGs (2052 hypermethylated and 390 hypomethylated CpGs) when compared to CI (360 hypermethylated and 66 hypomethylated CpGs). Comparison of these two lists of differently methylated CpGs revealed that 97.5 % of the CpGs showing aberrant methylation in CI were also deregulated in CII. In addition, we identified 691 CpGs containing no known SNPs that were differently methylated between the two clusters (671 hypermethylated and 20 hypomethylated CpGs) (Additional file 1: Table S4).

**Fig. 2:**
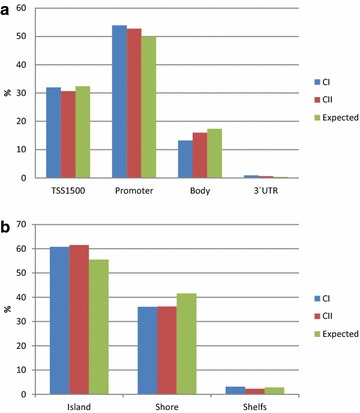
Distribution of the differently methylated CpGs across the gene annotations. This figure shows that DNA methylation signatures for tumors in cluster I and cluster II do not differ significantly with respect to the sequence context in which CpG methylation changes tend to occur; **a** TSS1500, promoter, body or 3′UTR and **b** Island, shore or shelf

**Fig. 3:**
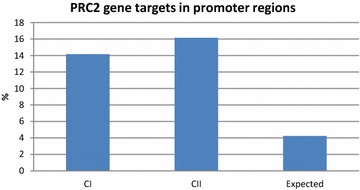
Percentage of cluster specific methylation events in promoter regions analyzed in terms of whether or not they have been identified as targets of the Polycomb group repressor complex 2 (PRC2). This figure shows that both cluster I and cluster II tumors show a higher percentage of PRC2 target genes in promoter regions than what expected just by change, being tumors in cluster II the ones sowing a higher percentage of PRC2 target genes

The resulting DNA methylation signatures for the two clusters were further analyzed in terms of functionally relevant DNA sequence elements. This analysis revealed that the distribution of the aberrantly methylated CpGs across the different gene annotations (TS1500, proximal promoter, body and 3′UTR of the gene) does not differ significantly between the two clusters and that they are not specially enriched for any of the gene locations (Fig. 2). In exploring the distribution of the methylation events across the CpG islands and their shores in the different gene locations, we found that both CI and CII involve a higher percentage of methylation event that what it was expected in CpG islands and a lower percentage in CpG poor regions, but that once more, the distribution of the methylation events across CpG islands was very similar between clusters (Fig. 2). We next measured the extent to which events involving CpG promoter methylation were enriched in Polycomb group repressor complex 2 (PRC2) target genes in each of the clusters. We found out that any of cluster specific signatures were significantly enriched as targets of the PCR2 in embryonic stem cells (p < 0.001), but this enrichment was slightly higher in CII compared to CI (Fig. 3).

### Selection of methylator cluster predictive biomarkers and validation of the markers

**Fig. 4:**
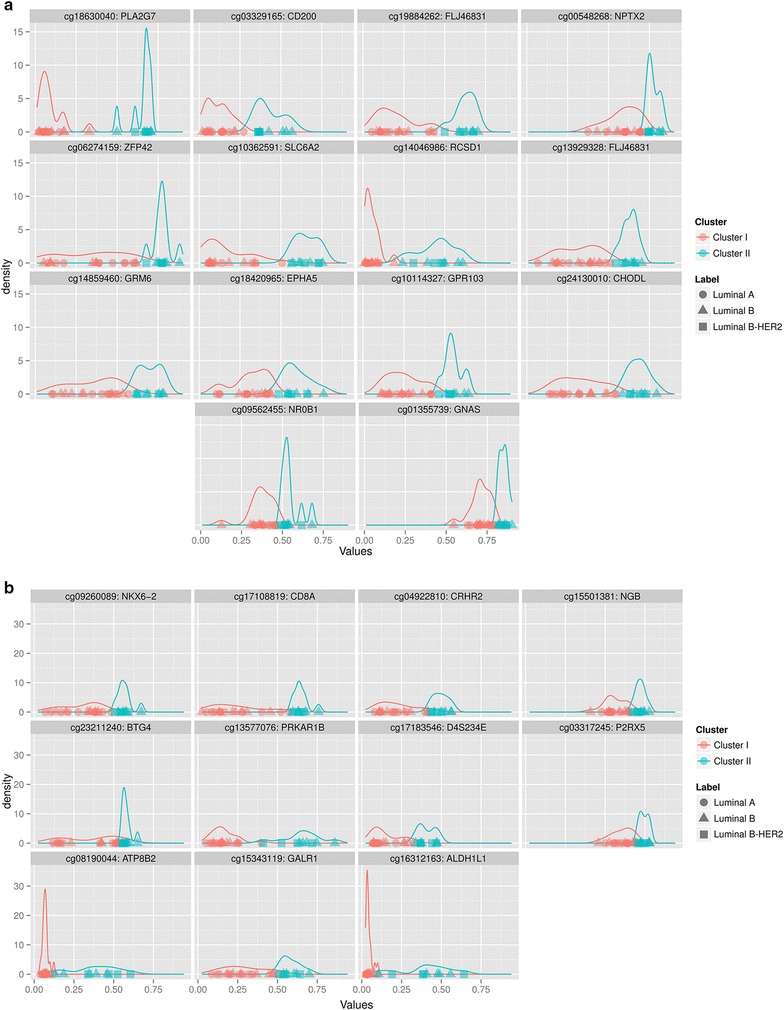
CpGs with a high cluster predictive power. **a**, **b** The 25 CpGs with the highest cluster predictive power

**Fig. 5:**
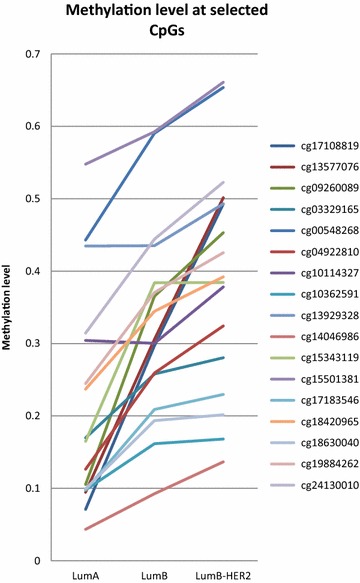
Methylation levels of the 17 CpGs with the highest cluster predictive power in the validation set (GSE31979). As in the discovery set Luminal A subtype showed the lowest methylation levels, luminal B-HER2 displayed the highest methylation values while luminal B tumors were associated with intermediate values

**Fig. 6:**
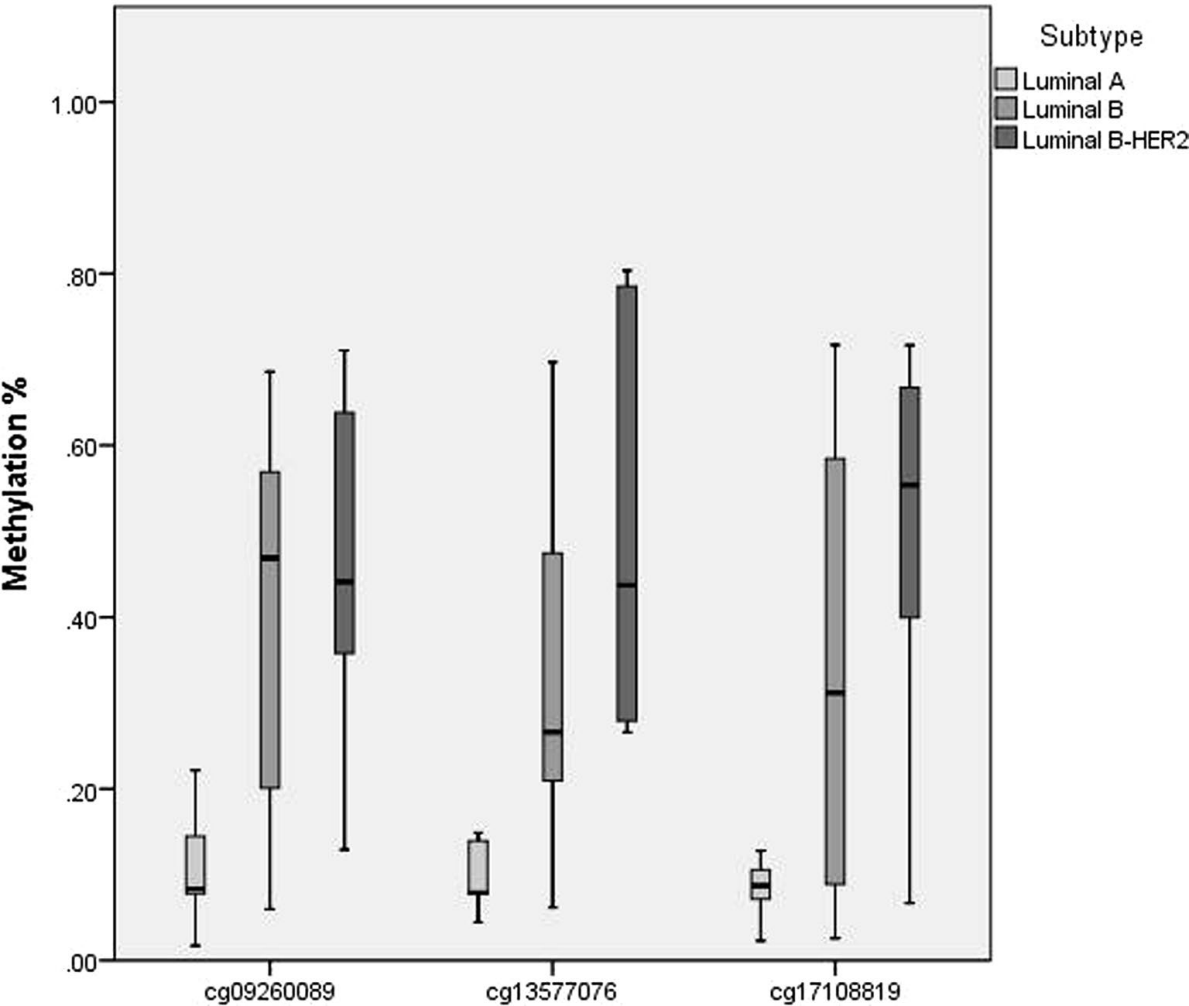
Boxplots of methylation levels of the three markers in the model in the validation set (GSE31979)

We next tried to identify CpGs markers that alone or in combination with other CpGs could classify luminal samples as CI or CII. We identify 25 CpGs among the 691 differently methylated between the two clusters whose cluster predictive power was absolute (Fig. 4a, b). These 25 markers were further validated in one publicly available dataset derived from 49 ER positive invasive breast carcinomas (GSE31979). Only 24 (5 luminal A, 7 luminal B-HER2 and 12 luminal B) of these 49 tumors had complete information about the expression patterns of ER, HER2, PgR and Ki-67 by immunohistochemical analysis, and thus could be subtyped using the same classification used by us (Fackler et al. 2011). We found that most of the selected loci (17 out of 25) followed the same patron observed in our discovery set, i.e. luminal A subtype showed the lowest methylation levels, luminal B-HER2 displayed the highest methylation values while luminal B tumors were associated with intermediate values (Fig. 5). Specifically, there were 3 specific CpGs out of these 17 (cg17108819, cg13577076 and cg09260089), that showed statistically significant differences between luminal A and luminal B-HER2 samples but did not show any difference between luminal Bs and the other two. These three CpGs that had almost no methylation overlap between the luminal A and luminal B-HER2 subtypes (Fig. 6) were further used as subtype predictive biomarkers. For each of these 3 CpGs, we measured mean value plus two times the standard deviation across the luminal A samples as cut-off value, and categorized samples as Epi-lumB-HER2 or Epi-lumA. Based on these classification, 100 % (5/5) of the luminal A tumors were classified as Epi-lumA, 100 % (7/7) of the luminal B-HER2 were classified as Epi-lumB-HER2 while 58 % (7/12) of the luminal B tumors were classified as Epi-lumB-HER2 and 42 % (5/12) as Epi-lumA. Comparison of the clinic-pathological features of the luminal samples clustered in Epi-lumB-HER2 and those clustered in Epi-lumA revealed that luminal Epi-lumB-HER2 presented higher stage (border significance, p value 0.1) as well as shorter Disease Free Survival (p value <0.05).

## Discussion

Luminal B breast tumors have aggressive clinical and biological features (Creighton 2012). As opposed to luminal A tumors, which usually receive endocrine therapy, they should be treated with a more aggressive therapy, which has not always demonstrated to be effective due to the molecular and clinical heterogeneity of this BC subtype (Pogue-Geile et al. 2013). In addition, discordance rates between the IHC-based surrogate classification schemes proposed so far for the luminal BCs and the multi-gene expression assays is still high, and the IHC-based surrogate definition of the luminal B subtype has been subjected to continuous modifications. Thus, identifying a surrogate classification scheme that stratifies luminal BCs in clinically meaningful subtypes has now became of paramount importance in the BC patient management. One of the latest and most accepted surrogate definitions has been the one proposed in the St Gallen International Breast Cancer Conference in 2013, upon which luminal A-like tumors are ER+, HER2−, with low Ki-67 expression (<20 %) and high PgR levels (≥20 %); luminal B-like (HER2-negative) tumors are ER+ and HER2− with high Ki-67 expression (≥20 %) or low PgR levels (<20 %); and luminal B-HER2 tumors are ER+ and HER2+, regardless PgR or Ki-67 expression. However, this new definition has already been questioned by some authors (Maisonneuve et al. 2014) and the surrogate definition is expected to be under further modifications. In this respect, we believe that a better characterization of the molecular alterations associated with of each of these new surrogate definitions would improve our understanding of the luminal tumor phenotype and led to a continuous refinement of the molecular classification of the BC.

From the epigenetic point of view, previous studies have documented that specific DNA methylation patterns can be related to some different luminal subtypes (Dedeurwaerder et al. 2011; Bediaga et al. 2010; Holm et al. 2010; Stefansson et al. 2015; Cornen et al. 2014; Li et al. 2014; Kamalakaran et al. 2011; Fang et al. 2011; Conway et al. 2014; Van der Auwera et al. 2010; Flanagan et al. 2010). Specifically, luminal B cancers have been usually associated with a methylator phenotype characterized by an extent DNA methylation in CpG islands (Bediaga et al. 2010; Holm et al. 2010; Stefansson et al. 2015). On the contrary, luminal A tumors have been described as being more heterogeneous in terms of their methylation patterns and with less methylation changes with respect to the normal (Bediaga et al. 2010; Stefansson et al. 2015). Aware of the biologic/epigenetic heterogeneity within luminal subgroup, we have compared for the first time the DNA methylation profiles associated the newly proposed IHC-based surrogate definition of the luminal B and compared it to that of the luminal A and B-HER2, thus providing new insights into the molecular features of the luminal B BCs. Our results, based on the unsupervised clustering of the luminal BC samples identified two significant DNA methylation groups. Sixty-two percent of the luminal B samples were grouped in cluster I (CI) together with luminal A tumors, while approximately third of the luminal B samples was clustered in cluster II (CII), which showed a higher proportion of methylation events and grouped the whole set of luminal B-HER2. Interestingly, tumors in CII were significantly bigger, had higher proliferative activity and worse outcome than those at CI. Further functional analysis of the epigenetic signatures associated with each of the two clusters indicated that distribution of the aberrant CpGs across the different gene annotations does not differ significantly for each cluster. On the other hand, when we investigated the extent to which genes with cluster specific methylation patterns were also PRC2 targets in ES cells, we found that both aberrant DNA methylation patrons in CI and CII were significantly enriched in PRC2 gene targets (i.e. they had a higher proportion of PRC2 than what is it expected), although CII showed a slightly higher enrichment in PRC2 target genes compared to tumors in CI. Finally, comparison of these two epigenetic signatures also revealed an extent overlap in the aberrantly methylated genes between the two cluster, i.e. 97.5 % of the CpGs showing aberrant methylation in CI were also deregulated in CII. Taken all together, our results indicated that (1) based on the St. Gallen IHC classification system, luminal B phenotype itself does not show a distinctive DNA methylation pattern (significant at AU > 95 %; by the pvclust method) but rather an heterogeneous methylation signature where 62 % of the samples clustered with luminal A and 30 % with the luminal B-HER2; (2) DNA methylation profiles enable the stratification of luminal B samples in two categories with differing epigenetic and clinical features, thus suggesting that adding certain epigenetic markers to the latest St. Gallen IHC classification scheme could improve the clinical relevance of the surrogate scheme; and (3) luminal B cancers have significantly greater numbers of methylation events than the luminal A, but methylation events in luminal A are present in luminal B, besides distribution of the methylation events across gene annotations is similar which all together may support the hypothesis that luminal B cancer precursors evolve from luminal A cancer/cancer precursors as has been previously speculated in Creighton et al. (2012).

A growing number of studies have identified gene hypermethylation profiles in subsets of luminal breast tumors (ER+) (Bediaga et al. 2010; Holm et al. 2010; Stefansson et al. 2015; Conway et al. 2014) and some of them have been independently associated with poorer clinical outcomes (Conway et al. 2014). Holm et al. (2010) described a signature of 196 CpGs that were found to be more frequently methylated in what they called lumB methylator phenotype. Bediaga et al. (2010) also identified a highly methylated luminal subtype characterized by the hypermethylation of a number of genes. On the other hand, Conway et al. (2014) reported four methylation-defined tumor clusters within the BC samples studied, among which cluster 3 (specially enriched in mRNA-subtype-luminal Bs) exhibited the highest methylation levels and worse long-term survival. Stefansson et al. (2015) also defined two DNA methylation-based subtypes, among which the Epi-LumB displayed a clear tendency to display CpG island promoter methylation events that was associated with unfavorable clinical parameters and reduced survival. Comparison of the above mentioned methylator signatures and the one described in the current study revealed that there is relatively high overlap and that there was a significant number of genes that were consistently deregulated (Additional file 1: Table S5), thus further supporting the existence of a luminal B methylator phenotype within the luminal B and luminal B-HER2 BCs.

In an attempt to create an epigenetic model that could characterize the luminal methylator phenotype described herein, we selected 25 CpG markers whose cluster prediction power was complete. We further analyzed the DNA methylation pattern of these 25 CpGs in an independent set of the luminal A, luminal B and luminal B-HER2 samples included in the GSE31979 study (Fackler et al. 2011) and found that most of them showed the same methylation patterns observed by us, i.e. high methylation levels in luminal B-HER2 and significantly lower ones in luminal A, with dispersed DNA methylation values in luminal Bs. Specifically, a methylation panel based on three CpGs (cg17108819, cg13577076 and cg09260089) grouped the entire set of luminal A samples as Epi-lumA and all the luminal B-HER2s as Epi-lumB-HER2. With respect to the luminal B tumors, 58 % of the luminal Bs were clustered with the luminal B-HER2 (i.e. displayed the luminal B methylator phenotype) while only 42 % were grouped with the luminal A. Further analysis of the clinical characteristics of the tumors displaying the luminal methylator phenotype revealed that these new DNA methylation subtype could be clinically relevant as indicated by their higher stage and worse outcome compared to those without the methylator phenotype. However, we note that the prognostic relevance of the methylator signature needs still to be further validated in independent and much larger luminal BC samples. On the other hand, it has recently been reported that some of the HER2-negative tumors could benefit from anti-HER2 therapy treatment (Pogue-Geile et al. 2013), thus it would be also interesting to know whether those luminal B-HER2 negative showing a methylator phenotype and similar epigenetic features to luminal B-HER2 respond to anti-HER2 therapy treatment.

## Conclusions

This work demonstrates that DNA methylation profiles enable the stratification of luminal B samples in two categories with differing epigenetic and clinical features. Specifically, a methylation panel based on three CpGs separates the entire set of luminal A samples from the luminal B-HER2s, and divides luminal B tumors in two groups clustering with luminal A and luminal B-HER2, respectively, suggesting that adding certain epigenetic markers to the latest St. Gallen IHC classification scheme could improve the clinical relevance of the surrogate scheme with respect to management of luminal B tumors.

## Additional file

10.1186/s40064-016-2235-0 **Table S1**: List of the 1261 differently methylated CpGs between tumors and adjacent tissue. **Table S2**: List of differently methylated CpGs in Cluster I. **Table S3**: List of differently methylated CpGs in Cluster II. **Table S4**: List of differently methylated CpGs between cluster I and II. **Table S5**: Comparison of the luminal B-methylator signatures described so far.

## Abbreviations

AU: approximately unbiased
AUC: the ROC curve
BC: breast cancer
CI: cluster I
CII: cluster II
FFPE: formalin fixed paraffin embedded
GEO: gene expression omnibus
IHC: immunohistochemistry
PRC2: polycomb group repressor complex 2
QC: quality control
